# Anatomy, Descriptive and Surgical

**Published:** 1859-11

**Authors:** 


ART. IV.—*Anatomy, Descriptive and Surgical.* By HENRY GRAY, F.R.S., Lecturer on Anatomy at St. George's Hospital. The Drawings by H. V. CARTER, M.D., late Demonstrator of Anatomy at St. George's Hospital. The Dissections jointly by the AUTHOR and DR. CARTER. With 363 Engravings on Wood. 8vo., pp. 754. Philadelphia: Blanchard & Lea, 1859.

WE can imagine with what diffidence the student of two or three centuries ago must have approached the ponderous folio which was to open out to him the mysterious secrets of his own organization, and to introduce him into a hitherto unexplored field of investigation. With what awe must its teachings have impressed him, as he perused the lengthy terms in Greek and Latin, or of classical derivation, which, when translated into familiar language, signified so little, while their sound implied such copiousness! The *embarras des richesses* must have been more keenly felt then than now, when so much more system is adopted in the classification of known anatomical facts. Many of the terms employed in the older works on anatomy have passed into disuse; although a large number still remain, occasionally perhaps shorn of their original dimensions. Anatomical works of the present day aim more at facilitating the student in the acquisition of a knowledge of his own structure, by offering him, when practicable, a terminology founded upon points of anatomical importance. The names of many of the muscles, for instance, are suggested by considerations of this nature, although other modes of nomenclature are likewise adopted. Whenever it is possible to excise from the older words superfluous syllables, the task is successfully accomplished; and although it may be true that such words as Basio-cerato-chondroglossus are stumbling-blocks in the path of the student, still the genius of the language tends to reform, and shorter words, which signify as much, are gradually replacing polysyllabic predecessors, which have become venerable only from their antiquity. We might wish, sometimes, that it were possible to modify entirely the old system of nomenclature, in order to found one based upon more rational views; but even in this mode it would be difficult to adopt any that would be universally recognized as the standard for all nations. Systematic works are tenacious, to a remarkable degree, of the legacies of words bequeathed to them by antecedent treatises; and naturally so, when we bear in mind that the completeness of a work is supposed often to depend, to a great extent, upon the amount of material embraced in its pages. These remarks, which apply occasionally to works on anatomy, where, however, copiousness of detail is unavoidable, apply more forcibly to other subjects of less importance.

Mr. Gray's work, like almost all other anatomical treatises, preserves many of these relics of antiquity, preferring, doubtless, to aim at a reputation for accuracy, rather than for originality—a quality, indeed, that a mere descriptive treatise cannot readily attain. The beaten track of description must be followed by every anatomical writer. Man, as he was in the infancy of the race, exists in bone, muscle, artery, the same now; and he who would describe his anatomy differently from his predecessors, can do so only by addressing the eye of the reader in a different way, or by varying from them in his command of language or his facilities in clothing simple facts in a more attractive garb.

A work on anatomy, to meet the wants of student, physician, and surgeon, must aim at the accomplishment of several important ends: to give a clear, concise statement of facts, which will aid the student at a time in his professional career when he feels most the need of its careful guidance; to furnish the physician with information on points which may be of vital consequence to him in his daily practice; and to indicate to the surgeon the varied and varying channels by which he may restore physical deviations, or the guiding marks by which he may know how to avoid important vessels or nerves, injury to which might destroy the life of his patient. We think the work of Mr. Gray meets these requirements, arranged as it is, throughout its pages, into divisions of topographical and descriptive anatomy, equally useful at any period of professional life. The great feature of the work is the mode of illustration. The cuts require no side-references or foot-notes, as in other anatomical treatises, the lettering of points to be studied in connection with each bone, muscle, nerve, etc. being a part of the engraving, and clearly marked. The cuts are of large size throughout; so much so, indeed, that the publishers in their catalogue notice state that "the large size of many of the illustrations prevents the selection of a favorable average specimen." We feel assured that the work must become popular with students and physicians for this reason, even if it had not other merits to recommend it.

We can plainly see that a work of this kind, printed page for page with the English edition, could have admitted of but little chance of rearrangement. As an example of slight but valuable reconstruction, however, we find, on comparing the English and American texts, the fifth nerve and its branches much more clearly divided in the republication than in the original work. We think the American editor has done wisely to separate "the ganglia connected with the fifth nerve" from the individual branches with which in reality they do not have an intimate relation, belonging rather to the "cephalic portion of the sympathetic system" than to the cerebro-spinal system of nerves under which they are described.

The author follows, in the divisions of his subject, the generally adopted routine course of the text-books on anatomy, which have been found the best adapted for the student; but furnishes, in addition, full and valuable remarks on surgical anatomy—an important branch, especially when presented, as in the work before us, in close connection with descriptive anatomy. Particular attention is paid in the first general division of osteology to the development of various parts of the bony structure, as well as the general outlines and prominent points of individual bones, dotted lines being added in the illustrations to show the origins and insertions of muscles, after the manner of Mr. Holden. The chapter on articulations occupies much more space than is usually given to this subject; and upwards of a hundred pages are devoted to the muscles and fasciæ. In the chapter on arteries, besides a careful description of vessels, particular attention is devoted to the surgical relations of every important artery, its peculiarities and varied modes of distribution, and the plan of operating, should ligation be required. The circle of Willis is beautifully shown in a full-page engraving, which would furnish a hint for a capital model of the base of the brain, and the distribution of vessels to that organ. The arteries of the pelvis are represented in a large cut; and the portal vein, with its branches, is exhibited in all its relations to intestine, liver, pancreas, stomach, etc.; an illustration which facilitates the physiologist in his efforts to explain the course of fluids—alcoholic liquors, for example—which pass into the abdominal venous system, and the pathological changes produced in this way in the structure and functions of the liver. The nervous system and organs of sense receive a large share of attention; and the system of illustration of that intricate part of the nervous apparatus—the brain—is novel, and exceedingly useful to the student.

After devoting several chapters to the full consideration of the organs of digestion, the thorax and thoracic viscera, the peculiarities of the fœtal circulation, the organs of voice and respiration, and the generative and urinary organs, Mr. Gray concludes his useful work with several chapters on the surgical anatomy of hernia, inguinal and femoral, and of the perinæum and ischio-rectal region.

The American edition of Mr. Gray's work has been carefully corrected, and contains but few typographical errors. In some of the statements we may differ with the author, but it is often in such minute anatomical distinctions of layers, etc., that we may fairly conclude that he has really and honestly described what he has seen, instead of always following implicitly the descriptions of others. In a second edition, the author would doubtless observe occasional faulty constructions of sentences, which were probably the results of hasty writing, but which add a little obscurity to the text. A hypercritical searcher after such faults of construction and typography, for the purpose of exposing every weak point, might, in any work, find abundant material for censure. If Mr. Gray deserves, more than other authors, condemnation for his errors, he may take refuge in the fact that the capital illustrations generally elucidate all such obscurities. We notice, occasionally, a variation between the text and the illustrations—in such words as "perineum" in the latter, and "perinæum" in the former, "gluteus" and "glutæus," etc.; but these are inconsistencies that it would have been difficult to avoid. In some of the illustrations, words containing *a* and *u* might sometimes be read incorrectly, as "mular," instead of "malar," from the occasional want of sharpness of the individual impression.

It is needless for us to suggest that the work before us might have contained subjects which it has omitted, or might have said less upon subjects which it very fully describes. There are a thousand matters directly or indirectly connected with anatomy, that might be introduced, and yet we think their introduction would scarcely be wise, although it might satisfy the craving of admirers of such topics. Pathology would lend hundreds of materials to construct the work, and to amplify it into a bulky volume, useless to the general student from its very size and fullness. The work has received no additions from American hands, although we are disposed to think that occasionally it might have been benefited by such an interposition. In such portions, for instance, as are rendered obscure from a faulty manner in which Mr. Gray expresses himself, an American editor, who had full power to add carefully, wherever deficiencies rendered it requisite, might have thrown considerable light.

There are many in the profession to whom the English edition of a book, with all its errors, is preferred to an American reprint, with all its improvements and modifications, apparently because English type and English paper are employed in the manufacture of the former and not of the latter. We do not bow to the omnipotence of this prejudice in the republication of Mr. Gray's book. The cuts are the same as in the London edition; the errors of the text, we are assured, have been corrected in many hundred instances, in spite of the author's acknowledgment, in his preface, of "the able assistance afforded him in correcting the proof-sheets in their passage through the press;" the table of contents and illustrations have been materially improved; and last, but not the least important feature of the work, the index has been more clearly arranged by spreading the headings over a larger surface, and its numerous errors of paging attended to. Wilson's Anatomy, so long a standard work in the medical schools, falls far behind Gray's work in copiousness of index; while Sharpey and Quain's Anatomy, although more full than the former, contains a very large number of subjects less than the latter.

We should recommend the work before us, in its improved American edition, as a useful book of reference to the unprofessional reader, who can see at a glance the illustrated representations of prominent points on which he desires information. To the general reader, as well as to the professional student and practitioner, we can speak confidently of the value of any standard book, whose illustrations seem to have been designed expressly to follow the text, and whose text follows very closely the illustrations. The getting up of the book in such beautiful style reflects credit upon the enterprising house which has given it an American circulation; and we trust that this edition, superior as it is in so many respects to the original work—superior, indeed, in some points to other anatomical works—may be widely disseminated through our country, as a means of diffusing more general anatomical information.

